# Pregnancy-related acute renal failure: A single-center experience

**DOI:** 10.4103/0971-4065.41283

**Published:** 2008-01

**Authors:** K. R. Goplani, P. R. Shah, D. N. Gera, M. Gumber, M. Dabhi, A. Feroz, K. Kanodia, S. Suresh, A. V. Vanikar, H. L. Trivedi

**Affiliations:** Department of Nephrology, Institute of Kidney Disease and Research Centre and Dr HL Trivedi Institute of Transplantation Sciences, Civil Hospital, Asarwa, Ahmedabad, Gujarat, India; 1 Department of Pathology, Institute of Kidney Disease and Research Centre and Dr HL Trivedi Institute of Transplantation Sciences, Civil Hospital, Asarwa, Ahmedabad, Gujarat, India; 2 Department of Sundaram Hospital, Chennai, Tamil Nadu, India; 3 Department of Pathology and Immunology, Institute of Kidney Disease and Research Centre and Dr HL Trivedi Institute of Transplantation Sciences, Civil Hospital, Asarwa, Ahmedabad, Gujarat, India

**Keywords:** Acute renal failure, renal cortical necrosis, pregnancy, sepsis

## Abstract

Pregnancy-related acute renal failure (ARF) is a common occurrence and is associated with substantial maternal and fetal mortality. It also bears a high risk of bilateral renal cortical necrosis. We conducted this study to evaluate the contributing factors and to assess the frequency of cortical necrosis. In this prospective study, of the 772 patients with ARF admitted at our institute between January 2004 and May 2006, 70 had ARF associated with pregnancy complications. ARF was diagnosed by documenting oliguria (urine output <400 ml/d) or mounting azotemia in the presence of normal urine output. (serum creatinine >2 mg%). Renal biopsy was performed if a patient was found to be oliguric or required dialysis support at the end of three weeks. The incidence of pregnancy-related ARF was 9.06%. Approximately 20% cases occurred due to postabortal complications in early pregnancy and 80% following complications in late pregnancy. Puerperal sepsis was the most common etiological factor in 61.42% of the patients. Preeclampsia accounted for 28.57% of ARF. Two-thirds of patients recovered with dialysis and supportive care. The incidence of biopsy proven renal cortical necrosis was 14.8% (10 of the 70 patients). The incidence of renal cortical necrosis was 28.57% in the early pregnancy group and 10.71% in the late pregnancy group. Postabortal sepsis was the most common precipitating event for renal cortical necrosis. Maternal mortality was 18.57%. Sepsis accounted for a majority of deaths (61.53%). Pregnancy-related ARF is common in western India. Puerperal sepsis is the most frequent etiological factor. Renal cortical necrosis is common and postabortal sepsis was the most common precipitating event. Sepsis accounted for a majority of maternal mortality.

## Introduction

Pregnancy-related acute renal failure (ARF) may comprise up to 25% of the referrals to dialysis centers in developing countries and is associated with substantial maternal and fetal mortality.^1^

In recent years, there has been a marked decline in the incidence of ARF associated with pregnancy; currently, cases that are severe enough to require dialysis occur in fewer than 1 in 20,000 pregnancies, although complications with transient mild to moderate glomerular filtration rate (GFR) decrease occur in approximately 1 in 8000 deliveries. The rate of septic abortion as the reason of the ARF was 33.3% in 1980-85 and has decreased to 6.3% in 1989-97.^2^

ARF in pregnancy is associated with a high risk for maternal mortality (9-55%).^3^

All factors that can cause ARF in a nonpregnant woman can theoretically cause renal failure in a pregnant woman, including volume depletion, bleeding and sepsis. Based on the stage of pregnancy, pregnancy-related ARF is divided into three groups, viz, first half, second half and postpartum ARF. Unskilled and septic abortion are the most common cause of ARF during the first half of pregnancy. During the second half, ARF is most commonly associated with preeclampsia or abruptio placentae. Postpartum renal failure is a specific entity and may be considered as a form of hemolytic-uremic syndrome occurring in the postpartum period.

ARF in pregnancy bears a high risk of bilateral renal cortical necrosis and consequently of chronic renal failure. Renal cortical necrosis is an uncommon entity and accounts for only 2% of all cases of ARF. Obstetric complications are the most common (50-70%) cause of renal cortical necrosis; abruptio placentae, septic abortion, preeclampsia, postpartum hemorrhage and puerperal sepsis are the conditions associated with pregnancy, and are responsible for renal cortical necrosis.^4^

The management of ARF following septic abortion or in the puerperium is similar to that of ARF in nonpregnant subjects, an exception being that hemorrhage may be concealed. This fact is often underestimated and even moderate blood loss can have deleterious effects on the kidneys. Therefore, blood should be replaced early.^5^

We undertook this study to evaluate the contributing factors responsible for pregnancy-related ARF, assess the frequency of cortical necrosis and acute tubular necrosis and factors contributing to their development.

## Materials and Methods

Between January 2004 and May 2006, 772 patients with ARF were admitted at the Institute Of Kidney Diseases and Research Centre and Institute Of Transplantation Sciences.

A total of 92 patients with ARF were pregnant. Of these, 22 patients had the evidence of renal disease prior to pregnancy and were excluded; hence, 70 patients with pregnancy-related ARF were studied.

Pregnant women who were healthy previously and had developed ARF were diagnosed in oliguria (Urine output <400 ml/d) and for mounting azotemia (Serum creatinine >2 mg%).

Exclusion criteria were the following:

1. Evidence of renal disease prior to pregnancy (glomerulonephritis, renal insufficiency from any cause)
2. History of hypertension or diabetes before gestation
3. History of renal stone diseases
4. Renal scarring on ultrasonography
5. Small size of the kidneys
6. Elevated serum creatinine prior to gestation

Thus, women with no history of oliguria or renal disease prior to gestation, normal-sized kidneys on ultrasound and no urological complication were included in the present study.

Detailed history, clinical examination and investigations were performed in all patients. Each patient underwent complete obstetric examination and removal of products of conception was performed as and when required. Specific inquiries were conducted regarding the mode of delivery, need for blood transfusion and surgical intervention. Renal biopsy was performed if a patient was oliguric or required dialysis at the end of three weeks.

Hemodialysis or peritoneal dialysis was performed according to standard indications.

Patients who became dialysis independent with good urine output and renal function were discharged and followed-up every fortnight for three months.

Statistical analysis was performed with unpaired *t* test. *P* < 0.05 was accepted as the level of statistical significance.

## Result

A total of 772 ARF patients were observed at the Institute Of Kidney Diseases And Research Centre and Institute Of Transplantation Sciences between January 2004 and May 2006; of these patients studied, 70 (9.06%) had pregnancy-related ARF.

The mean age of patients with pregnancy-related ARF was 25.6 years. The youngest patient was 20 years old and the eldest was 35 years old. In our study, 22 (31.4%) patients were primigravid and 48 (68.57%) were multigravid.

Fourteen patients (20%) presented in early pregnancy and five (7.14%), in late pregnancy. The major group was the puerperal group comprising 51 patients (72.85%).

A total of 45 patients (64.28%) had delivered in a hospital, while 11 (15.71%) delivered at home. Of these 11 patients who delivered at home, 4 died due to septicemia and disseminated intravascular coagulation (DIC), while renal function did not recover in 1 patient who had patchy cortical necrosis.

Twenty-five patients (35.71%) presented with anuria, while 44 (62.85%) with oliguria; only one patient had nonoliguric renal failure. Other presenting features were fever in 78.57%, edema in 72.85%, vaginal bleeding in 50% and dyspnea in 42.85%.

In 61.42% of the patients, puerperal sepsis was the most common etiological factor leading to ARF, while 32.85% of the patients had DIC on presentation. Hemorrhage as the etiology for ARF was present in 38.56% of the patients, APH in 14.28% and PPH in 24.28% of patients. Preeclampsia, eclampsia and HELLP syndrome accounted for 28.57% of patients with pregnancy-related ARF. Postabortal sepsis as a precipitating event for ARF was present in 20% of the patients [Table 1].

**Table 1:** Various etiological factors for pregnancy-related acute renal failure

Etiological factor	No. of patients (total = 70)	Percentage	Chugh *et al*. (total = 72)
Postabortion sepsis	14	20	43
PostMTP	11	15.71	
Spontaneous abortion	03	4.28	
Ante partum hemorrhage (APH)	10	14.28	5
Post partum hemorrhage (PPH)	17	24.28	7
Preeclampsia/eclampsia/HELLP	20	28.57	8
Puerperal sepsis	43	61.42	9
Malaria	03	4.28	
DIC	23	32.85	

A majority of the patients (97.14%) underwent hemodialysis, while one died without dialysis; further, one patient underwent peritoneal dialysis due to hypotension and died later. Peritoneal dialysis was initially performed in 8 patients (11.42%) due to initial hypotension, 7 patients among which improved and received hemodialysis later.

Evacuation of products used for conception was required in 15 patients (21.42%) and obstetric hysterectomy, in 4 (5.71%). One patient (1.42%) had perinephric pus collection that was drained.

Maternal mortality was 18.57%. Sepsis accounted for 61.53% (8 of the 13 patients) of deaths. Pulmonary edema was the cause of death in 2 patients (15.38%) who died within 24 h of admission. Two patients died due to HELLP syndrome and one (7.69%), due to hepatic encephalopathy.

Of the 81.42% surviving patients who were discharged, 54.28% had complete recovery of renal function; 12.85%, partial recovery; and 14.28% required chronic dialysis [Table 2].

**Table 2:** Maternal outcome

Maternal outcome	No. of patients (70)	Percentage
Survived	57	81.42
Complete recovery	38	54.28
Partial recovery	09	12.85
No recovery	10	14.28
Dead	13	18.57

**Fig. 1:**
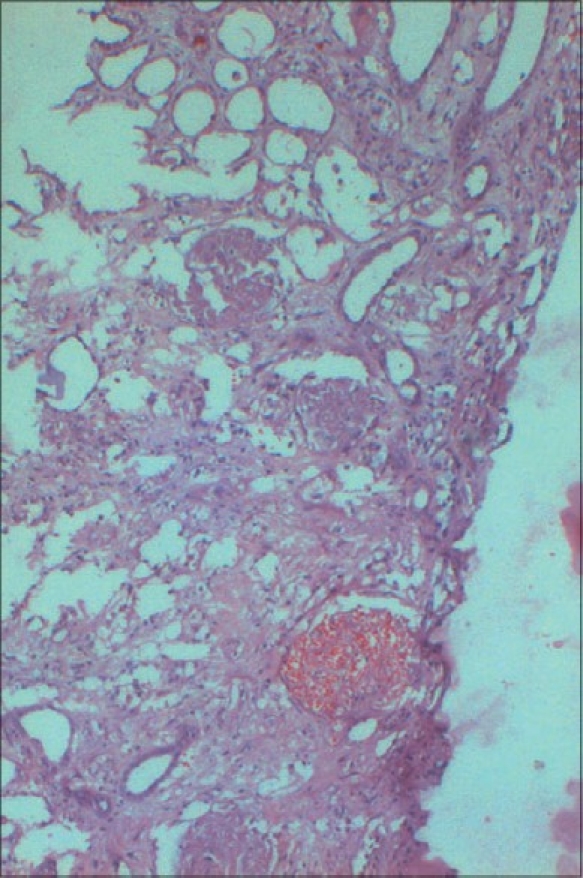
Photomicrograph showing diffuse necrosis in the renal cortex (H&E, ×150)

**Fig. 2:**
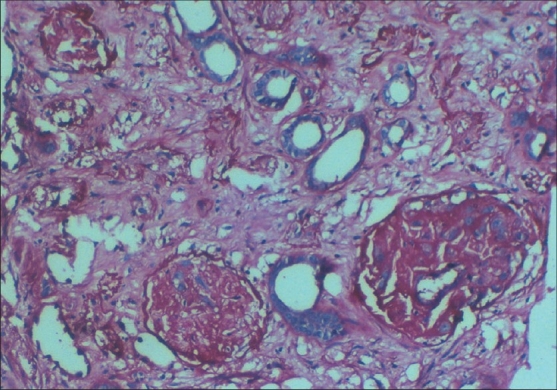
Shows fibrinoid necrosis of glomerular capillaries and ghost glomeruli with necrosis of all elements (H&E, ×450)

The incidence of cortical necrosis in pregnancy-related ARF was 14.28%. In the present study, renal biopsy was performed in 11 patients of which 10 had cortical necrosis and 1 had glomerular endotheliosis associated with preeclampsia [Figs. 1 and 2]. Of the 10 patients with cortical necrosis, 4 had developed the lesion in early (postabortal) pregnancy, while 6, in late pregnancy. The incidence of cortical necrosis was 28.57% in early pregnancy, while it was 10.71% in late pregnancy.

Factors affecting patient survival were sepsis, thrombocytopenia, DIC and liver involvement (*P* < 0.05), whereas the duration of dialysis and anuria on presentation were associated with poor renal survival (*P* < 0.05) [Table 3].

**Table 3:** Factors affecting outcome

Factors	Patient survival (*P* value)	Renal survival (*P* value)
Sepsis	0.036*	0.28
Thrombocytopenia	0.03*	0.3
DIC	0.0004*	0.14
Liver involvement	0.01*	-
Creatinine at presentation	0.09	0.3
Anuria	-	0.03*
Duration of dialysis	0.3	0.00036*

* *P* < 0.05 - Significant, DIC - Disseminated intravascular coagulation

Preeclampsia/eclampsia/HELLP was associated with ARF in 28.57% of the patients. Their mean age was 25 years, and 60% of the patients were multipara; 5%, renal cortical necrosis and 5%, glomerular endotheliosis on biopsy, both of which could not recover their renal function [Table 4].

**Table 4:** Etiology and course of pregnancy-related acute renal failure

Etiology	Incidence (%)	Mean age	Parity primi/multi (%)	Cortical necrosis (%)	Maternal mort (%)
Pre-edampsia/HELLP (3)	20/70 (28.57)	25.05	8/12 (40% / 60%)	1 + 1 (5)	3 (15%) 2 (66)
Antepartum hemorrhage	10/70 (14.28)	23.88	3/7 (30% / 70%)	1 (10)	2 (20)
Postpartum hemorrhage	17/70 (24.28)	27.05	5/12 (29.41% / 70.58%)	2 (11.76)	3 (17.64)
Post abortal sepsis	14/70 (20)	24.07	4/10 (28.57% / 71.42%)	4 (28.57)	1 (7.14)
Puerperal sepsis	43/70 (61.42)	26.20	14/29 (32.55% / 67.44%)	2 (4) (9.3)	2 (9) (20.93)

Approximately 20% of the patients had postabortal sepsis, 70% of which were multipara and 28.57% had cortical necrosis; maternal mortality in this group was only 7.14%.

Puerperal sepsis was present in 61.4% of the patients; 67% were multipara, cortical necrosis occurred in 9.3%, while maternal mortality was 20.93%. Positive blood culture was present in 9% of the patients. The organisms isolated were *Escherichia coli*, *Klebsiella* and *Pseudomonas*. Of the six patients with positive blood culture, four died (66.6%).

## Discussion

The incidence of pregnancy-related ARF in the developed countries is 1-2.8%. In the developing countries, the incidence is still remains at 9-25%, mostly due to late referral of pregnancy-related complications. In the present study, the incidence is 9.06%, which is similar to the results found in other literatures from India.^6^,^7^ The reason of the lower incidence in the developed countries is the prevention of the pregnancy complications and early and more effective treatment of preeclampsia. Septic abortion is not observed any more in developed countries.^3^

In the present study, 20% of the cases were due to postabortal complications in the early pregnancy, while 80% were in late pregnancy. This is in contrast with a previous study conducted in India in which 59.7% of patients were reported to have developed ARF in early pregnancy.^8^ This appears to be due to the legalization of abortion.

In our study, the incidence of cortical necrosis was 14.28%, while it was 23.8% in another study previously conducted in India.^6^ However, this rate is still higher than other causes of ARF which is <5%.^9^ The incidence of renal cortical necrosis was 28.57% in early pregnancy and 10.71% in late pregnancy. The higher incidence is probably due to late diagnosis and referral of complications related to dilatation and evacuation. In another study conducted in India,^9^ the incidence was nearly equal in the early (20.5%) and late (29%) pregnancies. This is in contrast to the western countries where postabortal ARF leading to renal cortical necrosis is rare (1.5%).^10^

In our study, the maternal mortality was 18.57%, while in a previous study conducted in India, it was approximately 30%.^11^ Kumar *et al*. recently reported a maternal mortality rate of 24%.^7^ This appears to be the result of aseptic delivery practices and early management of antepartum and postpartum hemorrhages.

## Conclusion

Pregnancy-related ARF is a common occurrence. Puerperal sepsis was the most common etiological factor responsible for pregnancy-related ARF. ARF due to postabortal sepsis is still a common complication. The incidence of biopsy-proven renal cortical necrosis is high with postabortal sepsis accounting for a significant number. Sepsis, thrombocytopenia, DIC and liver involvement were associated with maternal mortality. While the duration of dialysis and anuria was associated with renal survival. Maternal mortality is decreasing but sepsis is still accounted for a majority of deaths.
